# Effect of dietary counseling and dietary fiber on tolerability during weight management with EMP16 and conventional orlistat: a single-blind randomized pilot trial

**DOI:** 10.1038/s41598-026-62003-6

**Published:** 2026-07-13

**Authors:** Ulf Holmbäck, Stefan Grudén, Sandra Kuusk, Helena Litorp, Joakim Englund, Arvid Söderhäll, Göran Alderborn, Anders Forslund

**Affiliations:** 1 Department of Public Health and Caring Sciences, Clinical Nutrition and Metabolism, Husargatan 3, Box 564, Uppsala, 751 22 Sweden; 2 Empros Pharma AB, Solna, Sweden; 3 Clinical Trial Consultants AB, Uppsala, Sweden; 4 Department of Women’s and Children’s Health, Uppsala University, Uppsala, Sweden; 5 Department of Global Public Health, Karolinska Institutet, Stockholm, Sweden; 6 Department of Pharmaceutical Biosciences, Uppsala University, Uppsala, Sweden

**Keywords:** Dietary support, Fiber supplementation, Gastrointestinal tolerability, Diseases, Gastroenterology, Health care, Medical research

## Abstract

The aim of this trial was to assess whether dietary counseling together with supplemental fiber improves gastrointestinal (GI) tolerability during dose escalation with controlled-release orlistat–acarbose (EMP16) in adults who had reported prior GI side effects. In this randomized, single-blind, phase 2 pilot trial, adults with obesity (BMI ≥ 30 kg/m² or ≥ 27 kg/m² with risk factors) and prior GI problems to conventional orlistat or EMP16 were randomized 1:2 to EMP16 plus psyllium-based fiber or conventional orlistat plus maltodextrin placebo for 39 days, both with diet counseling. The primary endpoint was the composite gastrointestinal tolerability events (GITE) area-under-the-curve (AUC) score (diarrhea, oily spotting, flatulence with discharge, fecal incontinence) from daily electronic diaries. Additional endpoints included investigator-assessed GI adverse events, anthropometry, and cardiometabolic markers. Thirty-six participants completed treatment. GI event rates were much lower than expected in both arms, and GITE AUC differences were not statistically significant. Total events and time to first GI event favored EMP16 plus fiber. Greater relative weight loss occurred with EMP16 plus fiber vs. conventional orlistat plus placebo (p = 0.026), with similar ~ 10% LDL-cholesterol reductions. Dietary counseling during treatment with EMP16 or conventional orlistat may mitigate GI side effects in adults with prior GI side effects and co‑administration of fiber might further enhance tolerability and efficacy.

**Supplementary Information:**

The online version contains supplementary material available at 10.1038/s41598-026-62003-6.

## Background

Glucagon-like peptide-1 receptor agonists and multi-agonist therapies such as semaglutide and tirzepatide have transformed obesity management, producing weight loss of 15%–25% ^1^. However, real-world discontinuation rates remain high, affecting ~ 40% of patients within 3 months and 50%–65% by 12 months^1,2^, underscoring the need for a broader range of anti-obesity medications to support long-term adherence and maintained weight reduction.

EMP16 is a controlled-release (CR), fixed-dose combination of the lipase inhibitor orlistat and the α-amylase inhibitor acarbose and has been shown to achieve clinically relevant weight loss in two phase 2 trials^3,4^. In the initial dose escalation phases of these trials, a subset of participants in the EMP16 arms experienced problematic gastrointestinal (GI) related adverse events (AEs). Similar results have been observed in clinical trials studying orlistat and acarbose in their conventional dosage forms^5,6^. In contrast to other clinical weight management trials, very limited lifestyle and dietary support were provided to the participants in these trials, possibly affecting the prevalence of GI side-effects.

Previous research has reported GI side effects to be considerably lower when orlistat, in its conventional dosage form, was co-administered with fiber^7^. In the present pilot trial with EMP16 and orlistat in its conventional dosage form, we wanted to explore the impact of both individual dietary advice and use of supplemental dietary fiber on GI related AEs. The addition of fiber on top of dietary advice was hypothesized to decrease possible GI-related tolerability issues during the dose-escalation phase^7^.

## Materials and methods

### Study design

This was a randomized, single-blind, placebo-controlled phase 2 pilot trial. The trial included a screening period of up to five weeks and a 39-day treatment period and was conducted in Uppsala, Sweden, by an independent clinical research organization (CRO), Clinical Trial Consultants AB, Uppsala, Sweden. Authorization to conduct the trial was obtained in writing 22/04/2025 from the Member State through the Clinical Trials Information System (CTIS) (including competent authority authorization and an assessment by the Swedish Ethical Review Authority in Stockholm, Sweden) before the first participant was recruited (Application #5.1.1-2025-011918). Prior to any trial assessments, participants provided signed informed consent to participate in the trial. The trial was performed in accordance with the Declaration of Helsinki and ICH Good Clinical Practice, reported according to the Consolidated Standards of Reporting Trials (CONSORT) reporting guideline, and was registered in the EU Clinical Trials Register (EudraCT-nr 2024-520122-11-00).

### Participants

Participants were recruited using the CRO’s database and advertisements in social media platforms. The main eligibility criterion was prior experience of GI side-effects after treatment with EMP16 or conventional orlistat. Other key inclusion criteria were males or females aged ≥ 18 years; BMI ≥ 30 or ≥ 27 kg/m² in the presence of other risk factors; and no clinically significant abnormalities at the time of the screening visit, as judged by the Investigator. Full inclusion and exclusion criteria are listed in the supplemental information.

### Procedures

More detailed information is given in the supplemental information. Briefly, after screening, participants were randomized in a 1:2 ratio to two different arms:

- EMP16 + fiber: fixed dose combination of CR orlistat (120 mg) and CR acarbose (40 mg) plus Vi-Siblin^®^ S, a fiber supplement based on ispaghula seed coats, also known as psyllium husk.
- Active control + placebo: orlistat in its conventional dosage form (Alli^®^, 60 mg x 2) plus placebo dietary fiber (maltodextrin).

Blinding of participants was achieved by using identical capsules for EMP16 and active control, and identical bags for Vi-Siblin^®^ S and maltodextrin. After randomization, anthropometry assessments, and collection of fasting blood samples, participants were given the first doses of EMP16 + fiber or Active control + placebo. Participants self-administered the study treatment according to a dose-escalation schedule (Supplemental Table S1). At baseline, all participants received a 20-minute online advice by a nutritionist on proper healthy diet and strategies to ensure adherence. An online follow-up with the same nutritionist occurred 5 to 12 days after randomization, where the focus was implementation of diet strategies. Participants were also provided with access to a recipe database, containing suggestions for both meals and snacks. The dietary advice and the recipes were based on the Nordic Nutrition Recommendations^8^ with some minor adjustments (See Supplemental information for more information regarding the content of the advice). Details regarding assessment of compliance are given in the supplemental information.

### Outcomes

The primary endpoint was presence and level of discomfort of oily spotting, fecal incontinence, flatulence with discharge and diarrhea. The composite measure of these four domains was denoted gastrointestinal tolerability events (GITE). Participants recorded the presence of any GI tolerability events daily in an electronic diary (ViedocMe), approximately 2 h after dinner or at a time point considered more appropriate by the participant using four yes/no questions (with definition of events in parenthesis):

1. Have you had diarrhea today (liquid stools that made you have to go to the toilet more often than usual)?
2. Have you had oily spotting today (Have you seen oily spots in your underwear today? ).
3. Have you had flatulence with discharge today? (Have you had a “wet fart” today? )
4. Have you had fecal incontinence today? (Did you not make it in time to the toilet before having a bowel movement today? )

If an event occurred, the participants were asked to rate the level of discomfort caused by the event using a 7-graded Likert scale where 1 represented the most positive option (no discomfort at all) and 7 the most negative one (very severe discomfort), analogous to the Gastrointestinal Symptom Rating Scale (GSRS)^9^. At baseline, the participants were asked to report the presence and severity of GITE for the 3 days preceding the first dose. Secondary outcome variable was presence and severity of GI adverse events as judged by the PI (part of standard AE assessment). Exploratory variables included weight and body fat percentage measured using bio-impedance device (Tanita RD-545), height, waist circumference, fasting blood glucose, HbA1c, blood lipids and fatty liver index (FLI)^10^.

### Statistics

The power calculations were based on AE intensity recorded during the first 5 weeks in a previous trial^11^ using standard toxicity grading (Mild = 1, Moderate = 2, Severe = 3) multiplied with the AE duration since last visit. An effect size of 1 was assumed, based on a previous trial by Cavaliere et al. ^7^, where treatment with ispaghula seed coats improved orlistat tolerability with approximately 70%. With an assumed drop-out rate of 5%^11^, randomization of 39 participants was needed to achieve 36 evaluable participants. To increase power, as well as making the trial more pinpointed to participants experiencing initial side effects, only participants with prior problematic experiences (oily spottings, fecal incontinence or severe diarrhea) in conjunction with dosing of orlistat in its conventional dosage form or EMP16 were recruited.

The planned main model for the primary endpoint, mixed model for repeated measurements (MMRM), could not be used as the data was not normally distributed (See supplemental information). Instead, area-under-the curve for the whole trial period was calculated (GITE AUC) and non-parametric methods were employed (Mann-Whitney U test). Secondary endpoints were analyzed in the same way as the primary endpoint. The presence of GITE and GI AE was analyzed with Fisher’s Exact test. Exploratory endpoints including anthropometric parameters, fasting blood samples, and FLI are presented using descriptive statistics together with confidence intervals (CIs). Exploratory post-hoc analyses of time to first event (Log rank test) and relative weight loss (ANCOVA) were performed. Due to the exploratory and pilot character of the trial, hypothesis testing was one-sided using alpha 5%. Main analyses were done on the full analysis set (FAS) and supporting analysis was done on the per-protocol-set (PPS). Analyses were performed using Statistica version 14.2.0.18 (TIBCO, San Ramon, USA).

## Results

**Fig. 1:**
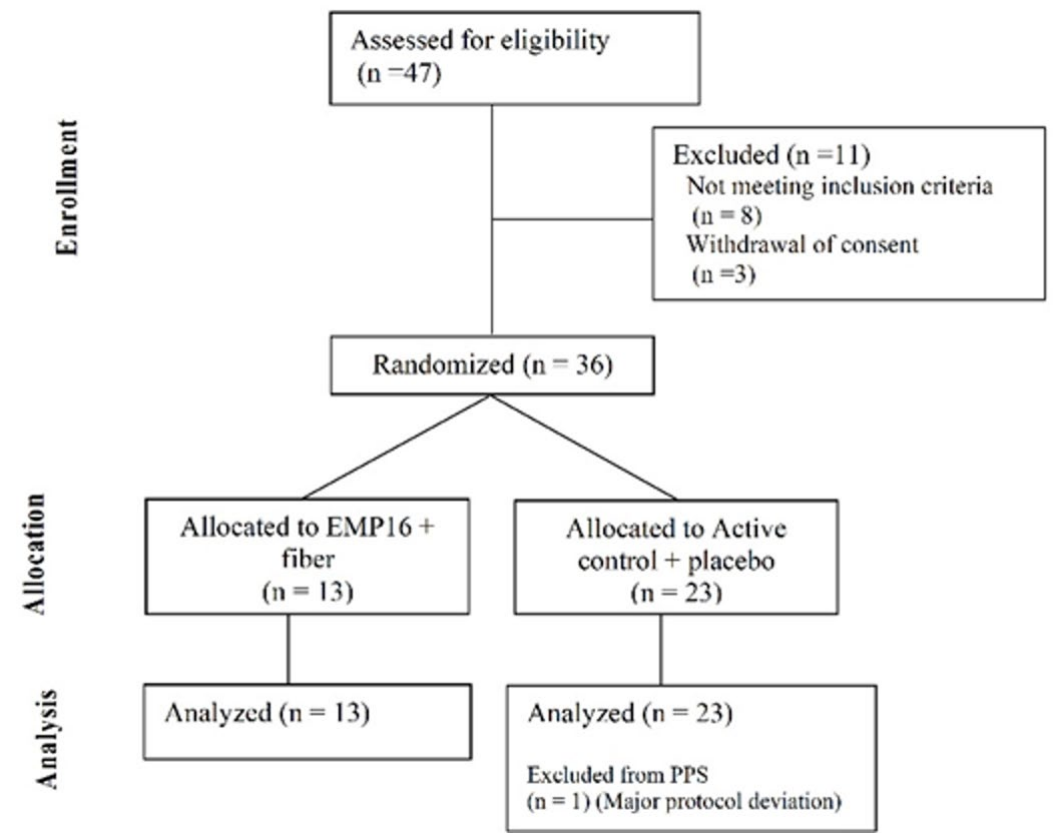
EMP16 + fiber, controlled release combination of orlistat/ acarbose combined with psyllium husk; Active control + placebo, orlistat in its conventional dosage form and maltodextrin placebo fiber.

A total of 47 potential participants were screened, of whom 36 were randomized and completed the trial (Fig. 1), baseline characteristics presented in Supplemental Tables S2 and S3.

High compliance was noted in both arms for both study drug as well as fiber supplement (supplemental tables S4 and S5).

In contrast to trial assumptions, participants in both arms reported very low GITE AUC score (both low presence of GI tolerability events and low rated level of discomfort) with no significant difference between the treatment groups (Table 1) when analyzing the FAS. Similar outcomes were seen in the PPS, but with clearer differences between EMP16 + fiber compared with Active control + placebo, where the diarrhea AUC score was significantly lower in the EMP16 + fiber arm (*p* < 0.05, Supplemental Table S7). There was very low GITE AUC score in both arms regarding oily spotting and fluctuance with discharge, with no significant difference between the arms (Table 1). Too few events of fecal incontinence occurred for statistical analysis. In the EMP16 + fiber arm, 3 participants reported one event each, with minor discomfort, moderate discomfort and moderate severe discomfort, respectively. In the active control+placebo arm, 2 participants reported one event each, with minor discomfort and moderate severe discomfort, respectively.

**Table 1:** Total GITE AUC and individual component AUC scores during the trial (FAS).

	EMP16 + fiber (*n* = 13)		Active control + placebo (*n* = 23)		
	Median^1^ (Q1, Q3)	Mean (SD)	Median (Q1, Q3)	Mean (SD)	*P*-value^2^
GITE	31 (3, 57)	39 (51)	29 (9, 92)	50 (47)	0.189
Diarrhea	6 (0, 30)	17 (22)	25 (6, 37)	30 (30)	0.075
Oily spotting	0 (0, 7)	8 (14)	2 (0, 8)	8 (14)	0.373
Flatulence with discharge	0 (0, 8)	14 (30)	2 (0, 9)	11 (24)	0.436

^1^ Median and mean AUC values were calculated from individual AUC for the whole trial. ^2^ Mann-Whitney U-test, one-sided.

The overall number of GI events was low, with a median of 9 GI tolerability events per participant over the whole trial for the EMP16 + fiber arm as compared to 11 events/participant in the Active control + placebo arm (Table 2). The few events that did occur were mostly rated as minor to mild discomfort (Supplemental Table S8). There were fewer total events and diarrhea events observed in the EMP16 + fiber arm compared with the Active control + placebo arm in the PPS (*p* < 0.05, Supplemental Table S9).

**Table 2:** Median and mean number of gastrointestinal tolerability events per participant during trial (FAS).

	EMP16 + fiber (*N* = 13)		Active control + placebo (*N* = 23)		
	Median (Q1, Q3)	Mean (SD)	Median (Q1, Q3)	Mean (SD)	*P*-value^1^
Total events	9 (1, 20)	12 (13)	11 (6, 27)	17 (14)	0.079
Diarrhea	2 (0, 7)	6 (7)	10 (2, 16)	10 (8)	0.070
Oily spotting	0 (0, 3)	2 (4)	1 (0, 3)	3 (5)	0.336
Flatulence with discharge	0 (0, 3)	4 (7)	1 (0, 4)	4 (8)	0.325

^1^ Mann-Whitney U-test, one-sided.

**Fig. 2:**
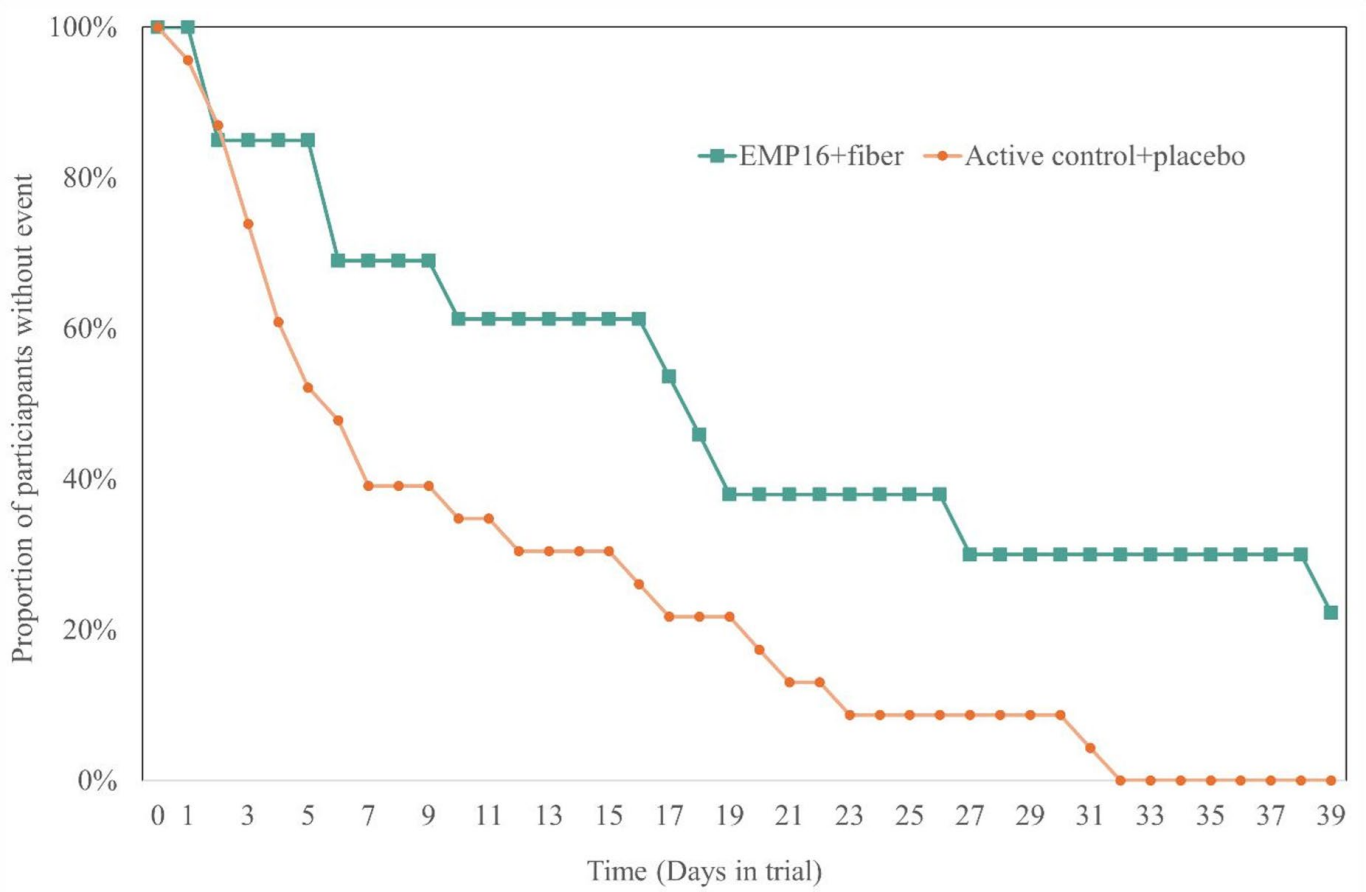
Proportion of participants without gastrointestinal tolerability events (GITE) in EMP16 + fiber (*N* = 13) and Active control + placebo (*N* = 23).

In the exploratory post-hoc analysis of the proportion of participants without GI events more participants in the EMP16 + fiber reported no GI events (*p* = 0.020; Table 3). As can be seen in Fig. 2, the time to the first GITE event was longer for EMP16 + fiber compared to Active control + placebo (*p* = 0.014).

**Table 3:** Participants with or without gastrointestinal tolerability events during the trial (FAS).

	EMP16 + fiber		Active control + placebo		*P*-value^1^
	Event	No event	Event	No event	
Total	10	3	23	0	0.020
Diarrhea	9	4	19	4	0.211
Oily spotting	6	7	13	10	0.365
Flatulence with discharge	6	7	13	10	0.365

^1^ Fisher Exact test, one-sided.

The standard AE assessment was partly in line with the GITE reporting, with numerically fewer, but not statistically significant, participants reporting any event of diarrhea in the EMP16 + fiber arm compared with Active control + placebo. Events of anal (fecal) incontinence and rectal discharge (oily spotting) were observed only through diary reporting, with no corresponding reports during routine AE assessments at trial visits (Table 4, Supplemental Table S10).

**Table 4:** Presence of gastrointestinal adverse events similar to GITE during the trial (FAS).

AE term^1, 2^	EMP16 + fiber (*N* = 13) *n* (%) m^3^	Active control + placebo (*N* = 23) *n* (%) m	*P*-value^4^
Anal incontinence (Fecal incontinence)	0%	0%	-
Rectal discharge (Oily spotting)	0%	0%	-
Diarrhea	5 (38%) 10	13 (56%) 22	0.2450

^1^ Dictionary derived term (reported term). ^2^ All events were rated as “mild” by the PI. ^3^ n, number of participants reporting event; (%, prevalence); m, number of events. ^4^ Fisher Exact test, one sided.

Weight and body composition improved in both arms, but with large intra-individual variation in especially waist circumference (Supplemental Table S11). As the CIs indicated a difference in relative weight loss between the treatment arms, an exploratory post-hoc ANCOVA analysis was performed, which confirmed that relative weight loss was larger in the EMP16 + fiber arm compared to Active control + placebo (*p* = 0.026).

Total cholesterol levels decreased in both arms, mainly driven by a clinically relevant ~ 10% reduction in LDL (Supplemental Table S11). As expected, HDL decreased in both arms. The CIs indicated that fasting glucose decreased slightly in both arms, and Fatty Liver Index only in the EMP16 + fiber group (Supplemental Table S11). No changes were observed in HbA1c and Gamma Glutamyl Transferase (GGT) (Supplemental Table S11).

Blinding of the trial seemed to be successful as no clear difference in willingness to continue taking the fiber supplement was observed between the arms (Supplemental Table S12).

## Discussion

The trial confirmed that dietary counseling, which also included diet adherence strategies based on the patient’s life situation, seems to have a major positive impact on tolerability in participants with prior GI problems associated with use of conventional orlistat or EMP16 when compared to previous trials The overall presence and level of discomfort of pre-selected GI events were lower than expected, with a highly skewed distribution, forcing a change in analysis method, and a resulting decrease in achieved power. The difference in GITE AUC score between treatment groups did not reach statistical significance but was suggestive of a positive effect of the added fiber in the EMP16 group. There were fewer GI events overall, more participants without any problematic GI events, and delayed time to first event in the EMP16 + fiber arm compared to the Active control + placebo arm, strengthening the observation of improved tolerability with the addition of fiber. In addition, relative weight loss was larger in the EMP16 + fiber arm than in the Active control + placebo arm.

Orlistat in its conventional dosage form is associated with GI side-effects such as oily spotting and diarrhea. Despite these side effects, and the rather modest weight loss, orlistat is still included in current weight management guidelines^12,13^, as it has been shown to have a favorable effect on blood lipids and diabetes prevention^5,14^. In addition to the recommendation to keep fat intake to ≤ 30E%^15^, various strategies have been shown to reduce the orlistat related side-effects, such as loperamide with or without simethicone treatment^16,17^, or addition of dietary fiber^7,18^. Apart from the recommendation to escalate the dose slowly^6^, there are, to our knowledge, no dietary interventions to mitigate the GI side-effects from acarbose (mainly flatulence and gastric distension), only. It has been shown that the proportion of carbohydrates, within 30% to 60% of total dietary intake, does not influence side effect profile^19^. Possibly a habitual diet with a high proportion of more complex carbohydrates facilitates adaptation to acarbose^20^.

In the present trial, several participants in the EMP16 + fiber arm reported flatulence and abdominal distension. These side-effects could come from either the acarbose component^6^ and/or the fiber supplement^21^. This could be seen as a shift from more “problematic” side-effects such as fecal incontinence and oily spotting to bloating and gas formation, which of course can also be inconvenient side-effects but arguably to a lesser degree. Although the dose of supplemental fiber was high with rapid dose escalation and no room for dose adjustments (unless PI decided dose reduction was necessary due to health considerations), the prevalence of bloating and gas formation was similar to previous trials^3,11,22^.

This trial did not aim to further study the impact of fiber on the side effect profile of orlistat in its conventional form; but instead explored if dietary fiber would alleviate GI side-effects in a selected patient population when being exposed to EMP16.

In the present trial, the difference in GITE AUC between EMP16 + fiber and the Active control + placebo was much smaller than anticipated. The low frequency of events does not seem to be due to incorrect enrollment of participants. Even though the overall number of GI events was low, > 60% of the Active control + placebo participants had recorded at least one GI event after only 5 days of treatment with one capsule per day. In contrast, in the trial by Cavaliere et al.^7^, underpinning the power calculation assumptions, the analogous GI score used was ~ 75% lower after treatment with conventional orlistat + fiber compared with conventional orlistat + placebo.

The most probable reasons for the unexpected low presence of events in the Active control + placebo group were the effect of the dietary advice and support given by the nutritionist as well as the selection criterion regarding previous experience of conventional orlistat or EMP16. In the trial by Cavaliere et al.^7^, the participants were given nutrition advice at baseline but with no follow-up, and the information was restricted to macronutrient composition and encouragement to remain physically active. In the present trial, the participants had a baseline meeting with a nutritionist, where not only dietary composition was discussed, but also strategies to implement the diet. The nutritionist reported that most participants had a good knowledge about what to eat and what not to eat; but needed more strategies to implement healthy habits. At the follow-up meeting, the outcome of the chosen strategy was discussed and adjusted if needed. In addition, the participants also had access to a recipe data base, with suggestions for meals and snacks, with appropriate fat content and focus on healthy carbohydrates, and overall in line with the Nordic Nutrition Recommendations^8^. Moreover, all participants in the present trial had previous experience of either conventional orlistat and/or EMP16. Possibly, recruited participants may have been more motivated and could perhaps more easily contextualize and individualize the dietary advice compared to the population enrolled in the Cavaliere trial^7^. Therefore, the added effect of fiber was somewhat diluted, compared to the Cavaliere trial^7^. Another factor potentially influencing the result could be the choice of placebo fiber. In the Cavaliere study^7^, rice flour was used as placebo, whereas maltodextrin was used in the present trial. Maltodextrin, apart from being a common food additive and easily obtainable in GMP quality, has been used as placebo to psyllium husk previously^23,24^, as it has a similar density, and a light sweetness resembling of the sorbitol addition in the marketed psyllium husk product used in the clinical study. However, maltodextrin consumed in larger doses and for longer duration may influence the GI system. Maltodextrin has for example been shown to decrease mucin production via increased intestinal inflammation^25^, which in turn decreases water content of stool and increases risk of constipation^26^. Possibly, the large volume of maltodextrin may have been sufficient to induce some degree of stool hardening, thereby influencing presence and/or rated discomfort level of GI events.

When comparing GITE diary with standard assessment of AE, there were comparable reports of loose stools/diarrhea in both arms. Moreover, the EMP16 + fiber arm reported abdominal distension and flatulence with similar frequencies as observed in a previously performed 6-month phase 2 trial^11^. However, there was a difference between diary reporting (GITE) and standard AE reporting in terms of presence of certain GI events. With standard AE questioning, no participants reported fecal incontinence or oily spotting, whereas using GITE, five events of fecal incontinence were reported, and 19 of the 36 participants reported at least one event of oily spotting. A difference between traditional AE reporting and GITE was not entirely unexpected as recollection differs when using closed end versus open questions^27,28^. GITE was collected using closed-ended, daily questions: “Have you experienced xxx?”; compared to the weekly open-ended questions used for assessing AE: “How have you been since we last saw each other or since you were last asked?”. Hence, a priori, there was an expectancy of higher frequencies of GITE events compared to AE events. This was a key reason to include the standard AE assessment, to enable comparisons to other trials.

Despite the short duration and small number of participants, a significant difference in relative weight loss between treatment arms was observed, in line with the previously mentioned phase 2 trial^11^. Furthermore, the ~ 10% lowering of LDL observed in the trial was larger than seen in much longer clinical trials in participants with similar characteristics using semaglutide^29^ or tirzepatide^30^. This clinically relevant lowering of LDL has also been shown in previous trials using EMP16^3,11^, and is a well-known effect of orlistat^31^. The lack of clinically relevant changes in fasting glucose and HbA1c was probably due to the absence of major glucose dysregulation in the participants and the short duration of the trial.

This explorative pilot trial was limited by a small, selected number of participants, short duration, and use of subjective outcome measures. Another limitation of this study is the use of one-sided tests at the 5% significance level, which is less stringent than the 2.5% one-sided level commonly applied in clinical trials. One-sided testing was prespecified because the research question was directional, with only improvement in GITE symptoms relative to the control considered clinically relevant. Nevertheless, some findings that reached significance at the 5% level would not have met a stricter threshold. As the primary endpoint was not met, statistically significant findings for secondary endpoints should be regarded as exploratory and interpreted with caution. Accordingly, these results should be viewed as hypothesis-generating rather than confirmatory. Moreover, a 1:2 allocation ratio was used instead of more commonly used ratios such as 1:1 or 2:1. This ratio was prespecified because the primary objective was to assess tolerability in a population enriched for susceptibility to gastrointestinal adverse events. Given the known association of the experimental treatment with such events, allocating a larger proportion of participants to the comparator arm, whose tolerability profile is well established, was considered appropriate to minimize unnecessary exposure to potential adverse effects. We acknowledge that this design yields less information on the experimental treatment than a 1:1 or 2:1 allocation. However, it reflects a balance between ethical considerations, patient safety, and the study objective. Still, the frequent interaction with the participants and their high compliance, together with the result from previous trials^4,7^, supports the findings. In the post-trial evaluation, there were no clear differences between the arms in willingness to continue to take the dietary fiber supplement, which may suggest that blinding was successful. Although the primary GITE AUC score comparison did not reach statistical significance due to fewer-than-expected events and limited power, consistent trends across multiple tolerability measures – including fewer GI events, more symptom-free participants, and delayed time to first event – might suggest a beneficial effect of fiber co-administration with EMP16.

## Conclusion

The trial confirmed that dietary counseling, which also included diet adherence strategies based on the patient’s life situation, seems to have a major positive impact on tolerability in participants with prior GI problems associated with use of conventional orlistat or EMP16 when compared to previous trials. Even though the added benefit of dietary fiber could not be statistically proven in the main outcome variable, the overall pattern suggests an improvement in tolerability, in line with previous research. The findings emphasize the importance of structured dietary counseling and behavioral support in mitigating side-effects during treatment, highlighting the need to consider diet implementation strategies, in addition to dietary composition, in future trials.

## Supplementary Information

Below is the link to the electronic supplementary material.

Supplementary Material 1

## Data Availability

The datasets used during the current study is available from the corresponding author on request.
