# Supplementary material for: Effect of dietary counseling and dietary fiber on tolerability during weight management with EMP16 and conventional orlistat: a single-blind randomized pilot trial
Source: Sci Rep. 2026 Jul 13;16:21835. doi: 10.1038/s41598-026-62003-6 (PMC13365554; doi:10.1038/s41598-026-62003-6)
Supplement: Supplementary file 1 — Supplementary Material 1 [file 41598_2026_62003_MOESM1_ESM.pdf]

Supplemental information

Effect of Dietary Counseling and Dietary Fiber on Tolerability during Weight Management with EMP16 and Conventional Orlistat: a single-blind randomized pilot trial

Ulf Holmbäck[1]*, Stefan Grudén[2], Sandra Kuusk[3], Helena Litorp[3,4,5], Joakim Englund[3], Arvid Söderhäll[2], Göran Alderborn[6], Anders Forslund[4]

[1] Department of Public Health and Caring Sciences, Uppsala University, Uppsala, Sweden

[2] Empros Pharma AB, Solna, Sweden

[3] Clinical Trial Consultants AB, Uppsala, Sweden

[4] Department of Women's and Children's Health, Uppsala University, Uppsala, Sweden

[5] Department of Global Public Health, Karolinska Institutet, Stockholm, Sweden

[6] Department of Pharmaceutical Biosciences, Uppsala University, Uppsala, Sweden

*Corresponding author: Ulf Holmbäck, Department of Public Health and Caring Sciences, Clinical Nutrition and Metabolism, Husargatan 3, Box 564, 751 22 Uppsala, Sweden.

+46(0)70 173 00 41, ulf.holmback@uu.se

Content

Material and Methods 4
Inclusion criteria 4
Exclusion criteria 5
Table S1 Dose-escalation schedule 7
Investigational medicinal products 7
Test product 7
Reference product (active control) 7
Active dietary fiber supplement 8
Placebo dietary fiber supplement 8
Randomization and blinding 8
Summary of the Nutrition Education Script 9
Participant disposition 11
Figure S1 Study Flow chart 12
Analysis of trial endpoints 12
Primary endpoint 12
Change of analytical model 13
Secondary endpoints 14
Exploratory endpoints 15
Treatment compliance 15
Results 16
Table S2 Baseline characteristics and demographics 16
Table S3 Treatment compliance IMP (FAS) 18
Table S4 Treatment compliance fiber supplement (FAS) 18
Table S5 Baseline anthropometry and fasting blood sample values 17
Table S6 Descriptive statistics, Gastrointestinal Tolerability Event (GITE) and its individual components. 19
Table S7 Total GITE score and the individual component AUC score (PPS). 20
Table S8 Likert scores during the trial, percentages of daily recordings of gastrointestinal tolerability events in both arms[1] 21
Table S9 Number of gastrointestinal tolerability events (Presence) during trial (PPS). 22
Table S10 Most frequent (>1 event in either arm) AEs by system organ class (SOC) and preferred term (PT) 23
Table S11 Changes in anthropometry and fasting blood samples, from baseline to end of study. 24

Table S12 Post-trial evaluation question “Would you be willing to keep on taking this fibre supplement in the long term?” (FAS) 25

References 25

Material and Methods

Inclusion criteria

For inclusion in the trial, potential participants had to fulfil the following criteria:

1. Were willing and able to give written informed consent for participation in the trial.
2. Had experienced GI tolerability issues (defined as the Presence of oily spotting, fecal incontinence and/or moderate/severe diarrhea as reported by the participant) in previous trials using EMP16 or had experienced corresponding GI tolerability issues using conventional orlistat, either in clinical trials or regular clinical treatment of obesity.
3. Males or females aged ≥18 years.
4. BMI ≥ 30 or ≥ 27 kg/m² in the presence of other risk factors based on participant interview e.g., hypertension (either or not treated with antihypertensive agents), glucose dysregulation (defined as elevated fasting glucose ≥6.1 mmol/L or glycated hemoglobin (HbA1c) >42mmol/mol), T2DM that is treated with lifestyle changes (no medication allowed), and/or dyslipidemia (either or not treated with antihyperlipidemic agents). If indicated, plasma/serum total cholesterol, low-density lipoprotein (LDL) cholesterol, high-density lipoprotein (HDL) cholesterol, and/or triglycerides (TG) could be measured to verify eligibility as judged by the Investigator.
5. No clinically significant abnormalities regarding physical examination, vital signs, electrocardiogram (ECG), and laboratory values at the time of the screening visit, as judged by the Investigator.
6. Adequate renal function: creatinine <1.5 times the upper limit of normal (ULN).
7. Adequate hepatic function: aspartate aminotransferase (AST), alanine aminotransferase (ALT), alkaline phosphatase (ALP) and gamma-glutamyl transferase (GGT) <2.5 times ULN and bilirubin <1.5 times ULN.

Exclusion criteria

Potential participants were not to enter the trial if any of the following exclusion criteria were fulfilled:

1. Regular use of any obesity medication within 1 month prior to Day 1 at the discretion of the Investigator.
2. Participants who were pregnant, who were currently breastfeeding, who intend to become pregnant within the period of the trial, or who gave birth within the 6 months preceding the screening visit.
3. T2DM treated with medication.
4. History of any clinically significant disease or disorder which, in the opinion of the Investigator, that either could have put the participant at risk because of participation in the trial or influenced the results or the participant's ability to participate in the trial including but not limited to:
    - GI problems/diseases, *e.g.* inflammatory bowel diseases and irritable bowel syndrome.
    - Cholestasis.
    - Chronical malabsorption syndrome.
    - History of severe allergic, cardiac or hepatic disease.
    - Previous GI surgery that might influence GI function significantly, such as previous bariatric surgery, and previous gallbladder surgery as judged by the Investigator.
    - Vitamin B12 deficiency or other signs of achlorhydria.

    Potential participants with well-treated chronic diseases (*e.g.*, celiac disease and lactose intolerance) could be included in the trial at the discretion of the Investigator.
5. Any clinically significant illness, medical/surgical procedure or trauma within 4 weeks of the first administration of investigational medicinal product (IMP).
6. Any planned major surgery within the duration of the trial.

7. Any use of drugs altering glucose metabolism and drugs used for diabetes (A10A and A10B) or drugs that were affected by, or that affect, orlistat and acarbose, within 2 weeks prior to the first administration of IMP.
8. Regular use of prescribed or non-prescribed medication within 2 weeks prior to the first administration of IMP as judged by the Investigator. Patients who were on stable treatment with anti-depressants (e.g., selective serotonin re-uptake inhibitors) for at least 2 months could be included at the discretion of the Investigator.
9. Untreated high blood pressure (systolic blood pressure >160 mmHg and diastolic blood pressure >100 mmHg at the screening visit).
10. Known hypersensitivity to any of the test substances.
11. Malignancy within the past 5 years, with the exception of *in situ* removal of basal cell carcinoma.
12. History of alcohol abuse or excessive intake of alcohol, as judged by the Investigator.
13. Presence or history of drug abuse and/or use of anabolic steroids, as judged by the Investigator.
14. Positive screening result for drugs of abuse or alcohol at the screening visit.
15. Any positive result at the screening visit for serum hepatitis B surface antigen, hepatitis C antibodies and/or human immunodeficiency virus.
16. Plasma donation within 1 month prior to screening or blood donation (or corresponding blood loss) during the last 3 months prior to screening.
17. Administration of another new chemical entity (defined as a compound which has not been approved for marketing) or had participated in any other clinical trial that included drug treatment within 3 months of the first administration of IMP in this trial. Participants who consented and screened but were not dosed in previous studies were not excluded.
18. The Investigator considered the *participant* unlikely to comply with trial procedures, restrictions and requirements.

Table S1 Dose-escalation schedule

Time period	Day 1–14	Day 15–28	Day 29–39
Number of capsules/day	1 (1 capsule at breakfast)	3 (1 capsule at each of the main meals)	6 (2 capsules at each of the main meals)
Daily dose orlistat/acarbose (mg) (EMP16 arm)	60/20	180/60	360/120
Daily dose of conventional orlistat (mg) (active control+placebo arm)	60	180	360
Daily dose of Vi-Siblin® S /placebo dietary fibre supplement	20 mL (8 g[1]) at breakfast	2 x 20 mL at breakfast and dinner	2 x 20 mL at breakfast and dinner

[1]The participants were provided with measuring cups and instructed on how to mix 20 mL (8 g) of Vi-Siblin® S /placebo dietary fiber supplement in fluid.

Investigational medicinal products

Test product

EMP16 is an FDC that contains 2 active pharmaceutical ingredients:

- Orlistat (CAS No. 96829-58-2)
- Acarbose (CAS No. 56180-94-0)

EMP16 was supplied as oral controlled-release (CR) capsules with the following strength:

- 60 mg CR orlistat/20 mg CR acarbose

Reference product (active control)

Orlistat in its conventional form was Alli® 60 mg in matching oral capsules. The target dose of Alli® was 120 mg orlistat.

Active dietary fiber supplement

Vi-Siblin® S is a granulate containing 88% ispagula seed coats, sorbitol and sodium chloride with a target dose of 40 mL (16 grams) per day. Vi-Siblin® S was supplied in plastic bags, containing 700 grams of Vi-Siblin® S.

Placebo dietary fiber supplement

Maltodextrin was used as a placebo as it has been used as placebo to psyllium husk previously [1, 2]. It has a similar density, and a light sweetness resembling of the sorbitol addition in the marketed psyllium husk product Vi-Siblin® S used in the present clinical study. Maltodextrin was supplied in plastic bags identical to bags used for Vi-Siblin® S, containing 700 grams of maltodextrin.

Randomization and blinding

On Day 1, participants were randomized in a 1:2 ratio to EMP16+fiber or active control+placebo. The randomization list was produced using SAS version 9.4 (SAS Institute, Inc., Cary, NC).

This was a single-blind trial. EMP16 and conventional orlistat (Alli®) were identical in appearance. Alli® capsules were recoated to match the appearance of the EMP16 capsules. Vi Siblin® S and placebo dietary fiber were provided in identical bags.

The use of a 1:2 ratio is unconventional, but as this trial was actively recruiting participants who experienced problematic GI side-effects after treatment with EMP16 or conventional orlistat, a higher than usual dropout rate in the active control+placebo arm was possible, despite the nutritionist dietary advice. The power calculation indicated that a possible higher dropout in the active control+placebo arm would still yield a power of around 80% with the current study design.

Summary of the Nutrition Education Script

This is an AI translated summary of the Swedish script used by the nutritionist when talking to the participants at the baseline. This script was also used when making a short movie that participants could use as a reminder.

“The material was designed to help participants develop healthier eating habits and improve long-term health, performance, and well-being. The overall message is that good nutrition supports not only physical health and weight management, but also mental focus, mood, energy levels, and quality of life.

A central theme throughout the program is balance. No food is completely off-limits, and no single food is healthy enough to be eaten exclusively. Success comes from making nutritious choices most of the time while allowing room for occasional treats. Participants are encouraged to establish regular meal patterns, which can help maintain satiety, support metabolism, and ensure adequate nutrient intake.

The recommended meal structure follows a simple plate model. Half of the plate should consist of vegetables, particularly fiber-rich options such as root vegetables, legumes, and cruciferous vegetables. One quarter should contain lean protein sources, including fish, poultry, eggs, beans, lentils, and lean meats. The remaining quarter should consist of high-fiber carbohydrates, preferably whole grains rather than refined alternatives like white rice or white pasta.

A major focus is increasing fruit and vegetable consumption. Participants are encouraged to consume at least 500 grams (about one pound) of fruits and vegetables daily. These foods provide vitamins, minerals, antioxidants, and fiber while contributing relatively few calories. Variety is emphasized, including fresh, frozen, cooked, and raw options. Vegetables such as kale, cabbage, root vegetables, peas, mushrooms, and peppers are highlighted as particularly nutritious choices.

The program also addresses sugar consumption and encourages participants to become aware of when and why they crave sweets. Limiting sugary foods can be achieved either by reducing portion

sizes or by reducing the frequency of consumption. Planning ahead, avoiding impulse purchases, and recognizing emotional eating triggers are presented as effective strategies.

When it comes to carbohydrates, the emphasis is on choosing nutrient-dense, high-fiber sources and reducing intake of highly processed, rapidly digested carbohydrates. Whole-grain breads, cereals, pasta, quinoa, oats, and other fiber-rich options are preferred over sweetened cereals, white bread, white rice, juice, and sugary yogurts.

Fat intake should be moderated, particularly from high-calorie sources such as processed meats, full-fat dairy products, fried foods, creamy sauces, and snack foods. However, healthy fats remain an important part of a balanced diet. The program encourages obtaining fats primarily from plant-based sources and fatty fish, including foods such as avocados, olives, nuts, seeds, and salmon.

Protein is highlighted as especially important for maintaining muscle mass, supporting metabolism, and promoting fullness. Lean protein sources such as chicken breast, fish, seafood, low-fat dairy products, beans, lentils, and eggs are recommended throughout the day. Protein-rich snacks and meals can help control hunger and reduce the likelihood of overeating.

The program provides practical examples of healthy snacks, including fruit with nuts, cottage cheese or Greek yogurt with berries, crispbread with cottage cheese and avocado, vegetables with hummus, and whole-grain bread with eggs. Water is presented as the preferred beverage, while sugary drinks, juice, alcohol, and other calorie-containing beverages should be limited.

Planning and preparation are described as essential factors for success. Participants are encouraged to identify situations in which they are most likely to make poor food choices and create systems that make healthy decisions easier. Strategies include meal planning, grocery shopping with a list, keeping nutritious foods readily available, and developing consistent routines for breakfast, lunch, snacks, and hydration.

Finally, the program recognizes that nutrition does not exist in isolation. Sleep and physical activity play important roles in weight management and overall health. Poor sleep can increase cravings for

sugary and high-fat foods, while healthy eating habits can support better sleep quality. Likewise, regular movement throughout the day is emphasized as more important than occasional intense workouts. Walking, cycling, taking the stairs, and reducing sedentary time are presented as simple yet highly effective ways to improve health and support long-term weight management.”

Participant disposition

The trial was conducted at single site in Uppsala Sweden. A total of 47 potential participants were screened, with 8 screen failures and 3 withdrawals of consent before randomization (Supplemental Figure S1). Thirty-six participants were randomized and completed the trial.

According to the power calculation, 39 participants were needed to ensure 36 evaluable participants. In a first enrollment period, 26 participants were randomized and started the trial in June 2025. All 26 completed the trial in July. A second enrollment period started in August 2025 and 13 additional participants were enrolled. When 3 out of these 13 enrolled participants withdrew their consent before randomization, it was decided not to replace these 3 late withdrawals. The two main reasons were:

1) that there were no indications that these three could be replaced within a reasonable time, as the screening and enrollment processes turned out to be much more challenging than anticipated;

2) There were no withdrawals from the initial 26 randomized patients so the sponsor judged that the possibility of ending up with 36 evaluable participants was sufficiently high even if total randomization was less than 39 participants.

One of the participants in the active control+fiber arm was no included in the PPS due to major protocol deviation (missed four evening doses of placebo fiber intake at the end of the trial).

Figure S1 Study Flow chart

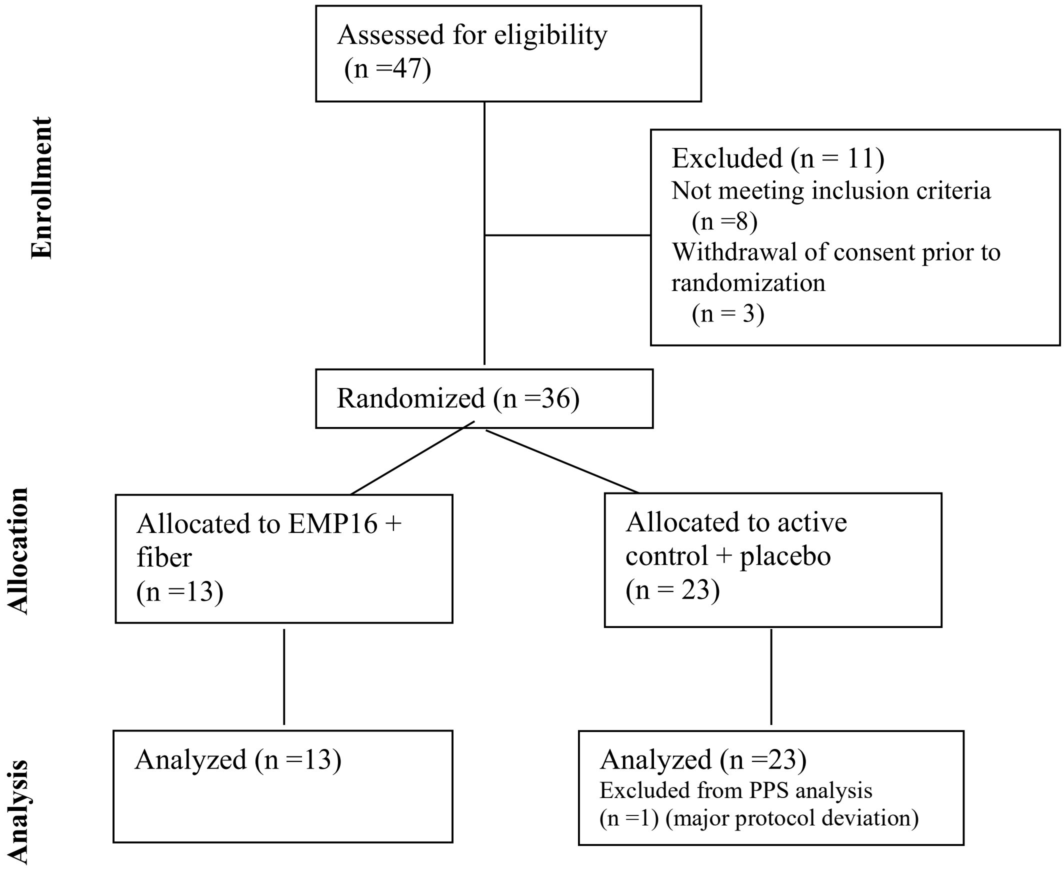

Analysis of trial endpoints

Primary endpoint

The primary outcome variable, gastrointestinal tolerability events (GITE) score was derived by summing up the presence and level of discomfort from the individual Likert scales (oily spotting, fecal incontinence, flatulence with discharge and diarrhea) per day – inferring a composite intensity score of 0 to 28 per participant and day. In this derivation, if a patient reported that he/she had no GITE for a particular day, the total GITE score was set to 0 for that day. If data for a particular participant and day was missing, the value was imputed by using interpolation between observed data points, providing the missing data point was in the middle of the participant's observed data

series. If the missing data point was at the beginning of the observed data series, imputation was based on first valid observation. The imputation was performed for each Likert scale separately. Subsequently, a Likert area under the curve (AUC) was calculated from the daily total GITE score as follows:

1. In case of participant had dropped out of the trial, the last non-missing composite Likert score (providing such exists) was carried forward to all subsequent days, implying a conservative imputation approach with regard to this scenario.
2. Secondly, an AUC was calculated for each participant using the linear trapezoidal rule according to the formula:

$$\sum_{k=1}^{N} \delta t \times \frac{L_{k-1} + L_k}{2}$$

where t = time, L = composite Likert score, $\delta t = t_k - t_{k-1}$. If the composite Likert score was missing for a particular participant and day, but there is a non-missing score before and after the missing timepoint, the formula above was adjusted to implicitly impute the missing value using interpolation between observed data points.

Change of analytical model

The planned main model for analysis of the primary endpoint was a mixed model for repeated measurements (MMRM) with derived weekly total GITE score as the dependent variable and timepoint (week 1, week 2, etc.), treatment and the interaction timepoint*treatment as the fixed categorical effects, the mean of the baseline total GITE score as a continuous covariate and participant nested within treatment as a random effect.

1. As all but one participant scored 0 in GITE at baseline, baseline GITE was not entered as a co-variate into the model.
2. When performing a fixed model for repeated measurements and without participant nested within treatment as a random effect, analyses of residuals showed clearly that normality

could not be assumed. This can also be seen in the distribution shown in supplemental table It was concluded that using a mixed model for repeated measurements with participant nested within treatment as a random effect, would not affect the outcome.

Therefore, the planned main analytical model could not be used.

The resulting AUC was summarized descriptively and analyzed using non-parametric methods. Differences in GITE AUC between EMP16+fiber and active control+placebo were tested using the Mann-Whitney U-test. Area under the curve was also calculated for the individual components of the GITE, using the above-described procedure including Mann-Whitney U-tests. However, since only 5 fecal incontinence events were reported in the whole trial, these events are merely listed separately and AUC was not calculated.

In addition, as differences in number of GI events were observed between EMP16+fiber and active control+fiber, an explorative analysis was performed using a Kaplan-Meier plot with right censoring in order to graphically display the proportion of participants who had not experienced any event, which is analogous to an analysis of the time to the first GITE event. The difference between the time to event curves for EMP16+fiber and active control+placebo was investigated using a Log rank test.

Secondary endpoints

The presence of GI-related AEs using standard AE reporting (Mild=1, Moderate=2, Severe=3, as rated by the Investigator) or GITE events reported by the participants (two different sets of analyses) for the whole treatment period was analyzed for each symptom (oily spotting, flatulence with discharge and diarrhea), as well as all symptoms jointly, using Fisher's exact test to exploratively assess statistical differences between the trial arms. In this analysis, a participant was set to "Yes" if they experienced the symptom anytime during the treatment period and "No"

otherwise. Differences in number of events of GITE between EMP16+fiber and active control+placebo, as well of presence of diarrhea, flatulence with discharge and oily spotting, were tested using Mann-Whitney U-test.

Exploratory endpoints

Anthropometric parameters, fasting blood samples and Fatty Liver Index (FLI)[3] were presented using descriptive statistics together with CIs to investigate differences in each treatment arm compared to baseline (within groups assessments). As CIs indicated a possible difference in relative weight loss, an exploratory analysis of co-variance (ANCOVA) was performed, comparing relative weight loss between the arms, using baseline weight as co-variate.

Treatment compliance

Participants self-administered EMP16/conventional orlistat at home. The first dose of EMP16/conventional orlistat was taken at the clinical site, together with the first dose of Vi-Siblin® S/placebo dietary fiber supplement.

The number of capsules handed out at each visit was documented in the eCRF. The number of returned capsules was counted at each visit to the clinic and documented in the eCRF. Text messages, reminding all participants to bring all EMP16/conventional orlistat bottles (including empty bottles) were sent prior to each visit. The dates for the first and last dose, respectively, were recorded in the eCRF.

IMP compliance was calculated as follows:

$$compliance\ (\%) = 100 * \frac{number\ of\ delivered\ capsules - number\ of\ returned\ capsules}{expected\ number\ of\ used\ capsules}$$

Compliance calculations was based on the individual treatment period. The number of capsules handed was documented in the eCRF. The number of returned capsules was counted at each visit to the clinic and documented in the eCRF. If a participant failed to return any empty containers, these

containers was considered to be unused when calculating compliance (resulting in worse overall compliance).

Compliance of Vi-Siblin® S/placebo dietary fiber supplement was addressed using the eDiary for GITE reporting, see Section 0.

Vi-Siblin® S/placebo compliance was calculated based on the participant's own registrations in the eCRF as follows:

$$compliance\ (\%) = 100 * \frac{number\ of\ "yes"\ when\ asked\ if\ fiber\ has\ been\ taken}{number\ of\ times\ asked}$$

Results

Table S2 Baseline characteristics and demographics

**Assessment**	**EMP16+fiber (N=13)**	**Active control+placebo (N=23)**
Age [years, mean (SD)]	56 (9)	51 (10)
Sex		
Female	8/13 (62%)	17/23 (74%)
Male	5/13 (38%)	6/23 (26%)
Ethnicity		
Hispanic or Latino	0/13 (0%)	3/23 (13%)
Not Hispanic or Latino	13/13 (100%)	20/23 (87%)
Race		
Black or African American	0/13 (0%)	3/23 (13%)
White	13/13 (100%)	20/23 (87%)

Table S3 Baseline anthropometry and fasting blood sample values

Variable	EMP16+fiber (N=13)	Active control+placebo (N=23)
Weight (kg)	101.3 (92.1 to 110.5)	103.5 (97.6 to 109.3)
Body Mass Index (kg/m^2)	35.4 (32.8 to 38.0)	35.8 (33.6 to 38.0)
Body fat %	41.0 (36.9 to 45.1)	42.0 (38.5 to 45.4)
Waist Circumference (cm)	116.5 (110.2 to 122.7)	115.6 (110.3 to 120.9)
Total cholesterol (mmol/L)	5.62 (5.19 to 6.04)	5.03 (4.66 to 5.39)
HDL cholesterol (mmol/L)	1.33 (1.20 to 1.45)	1.33 (1.21 to 1.46)
LDL cholesterol (mmol/L)	3.89 (3.51 to 4.28)	3.20 (2.88 to 3.52)
Triglycerides (mmol/L)	1.58 (1.38 to 1.79)	1.33 (1.03 to 1.62)
HbA1c (mmol/mol)	34.69 (33.50 to 35.89)	35.26 (34.00 to 36.53)
Glucose (mmol/L)	5.95 (5.72 to 6.17)	5.68 (5.51 to 5.86)
GGT (uKat/L)	0.63 (0.51 to 0.75)	0.55 (0.37 to 0.73)
FLI[1]	90.5 (85.8 to 95.2)	86.14 (81.8 to 90.5)

[1] FLI, Fatty liver index, was calculated from BMI, waist circumference, TGs and GGT [3]); GGT, gamma glutamyl transferase.

Table S4 Treatment compliance IMP (FAS)

Assessment (unit)		EMP16+fiber (N=13)	Active control+placebo (N=23)	Total (N=36)
Compliance (%)	Mean (SD)	101 (2)	100 (3)	101 (3)
	Median (Q1, Q3)	102 (100, 103)	101 (98, 103)	101 (99, 103)
	Min, Max	99, 103	93, 103	93, 103

Table S5 Treatment compliance fiber supplement (FAS)

Assessment (unit)		EMP16+fiber (N=13)	Active control+placebo (N=23)	Total (N=36)
Compliance (%)	Mean (SD)	100 (1)	98 (3)	98 (3)
	Median (Q1, Q3)	100 (100, 100)	100 (97, 100)	100 (98, 100)
	Min, Max	98,100	87, 100	87, 100

Table S6 Descriptive statistics, Gastrointestinal Tolerability Event (GITE) and its individual components.

	**GITE**	**Diarrhea**	**Oily spotting**	**Flatulence with Discharge**	**Fecal Incontinence**
EMP16+fiber (N=13)					
Median	31	6	0	0	0
Q1	3	0	0	0	0
Q3	57	30	7	8	0
Min	0	0	0	0	0
Max	183	68	49	103	5
Mean	39	17	8	13	0.9
SD	49	21	14	28	1.7
Active control+placebo (FAS, N=23)					
Median	29	25	2	2	0
Q1	15	7	0	0	0
Q3	77	36	8	9	0
Min	1	0	0	0	0
Max	145	100	58	98	5
Mean	50	30	8	11	0.3
SD	46	29	14	24	1.2
Active control+placebo (PPS, N=22)					
Median	29	27	2	2	0
Q1	12	8	0	0	0
Q3	84	37	8	7	0
Min	1	0	0	0	0
Max	145	100	58	98	5
Mean	51	32	8	11	0.4
SD	47	29	14	24	1.2

Table S7 Total GITE score and the individual component AUC score (PPS).

	EMP16+fiber (n=13)		Active control+placebo (n=22)		
	Median[1] (Q1, Q3)	Mean (SD)	Median (Q1, Q3)	Mean (SD)	P-value[2]
GITE	31 (2,5, 57)	39 (51)	29 (12, 84)	51 (47)	0.176
Diarrhea	6 (0, 30)	17 (22)	27 (8, 37)	32 (29)	0.049
Oily spotting	0 (0, 6,5)	8 (14)	2 (0, 8)	8 (14)	0.325
Flatulence with discharge	0 (0, 7.5)	14 (30)	2 (0, 7)	11 (24)	0.496

[1] Median and mean values were calculated from individual AUC for the whole trial.

[2] Mann-Whitney U-test, one-sided.

Table S8 Likert scores during the trial, percentages of daily recordings of gastrointestinal tolerability events in both arms[1]

	**Diarrhea**		**Oily spotting**		**Flatulence with discharge**	
	EMP16[2]	AC	EMP16	AC	EMP16	AC
No event	86.0%	74.9%	93.5%	92.5%	90.3%	89.3%
No discomfort	0.0%	0.9%	0.2%	0.3%	0.2%	0.3%
Minor discomfort	2.2%	5.5%	1.2%	2.7%	1.0%	3.7%
Mild discomfort	7.7%	9.7%	3.4%	3.2%	3.6%	5.5%
Moderate discomfort	3.6%	7.2%	1.4%	1.1%	2.4%	1.1%
Moderate severe discomfort	0.4%	1.6%	0.4%	0.1%	2.0%	0.1%
Severe discomfort	0.2%	0.2%	0.0%	0.0%	0.6%	0.0%

[1] Calculated as number of events with a particular score divided with total number of possible events. For example, in the active control + placebo arm, 87 events were recorded as "mild discomfort" for Oily spotting. Total possible events = 23 subjects * 39 days = 897. 87/897=3.4%.

[2] EMP16, EMP16 + fiber; AC, Active control + placebo

Table S9 Number of gastrointestinal tolerability events (Presence) during trial (PPS).

	**EMP16+fiber (N=13)**		**Active control+placebo (N=22)**		
	Median (Q1, Q3)	Mean (SD)	Median (Q1, Q3)	Mean (SD)	P-value[1]
Total events	9 (1, 20)	12 (13)	11 (6, 27)	17 (14)	0.074
Diarrhea	2 (0, 7)	6 (7)	10 (2, 16)	10 (8)	0.046
Oily spotting	0 (0, 3)	2 (4)	1 (0, 3)	3 (5)	0.301
Flatulence with Discharge	0 (0, 3)	4 (7)	1 (0, 4)	4 (8)	0.375

[1] Mann-Whitney U-test, one-sided.

Table S10 Most frequent (>1 event in either arm) AEs by system organ class (SOC) and preferred term (PT)

TEAEs by MedDRA SOC and PT	EMP16+fiber (N=13) n (%) m[1]	Active control+placebo (N=23) n (%) m
**Gastrointestinal disorders**	**12 (92%) 27**	**16 (70%) 43**
Diarrhea	5 (38%) 10	13 (56%) 22
Flatulence	8 (61%) 8	3 (13%) 3
Abdominal distension	4 (31%) 4	2 (9%) 2
Infrequent bowel movements	2 (15%) 2	1 (4%) 1
Steatorrhea	0	3 (13%) 4
Abdominal pain upper	1 (8%) 1	2 (9%) 3
Constipation	1 (8%) 1	1 (4%) 2
Feces hard	0	1 (4%) 2
**Infections and infestations**	**2 (15%) 2**	**6 (26%) 6**
Nasopharyngitis	2 (15%) 2	5 (22%) 5
**Nervous system disorders**	**3 (23%) 3**	**7 (30%) 10**
Headache	3 (23%) 3	7 (30%) 10
**General disorders & administration site conditions**	**2 (15%) 2**	**1 (4%) 1**
Pyrexia	2 (15%) 2	1 (4%) 1

MeDRA, Medical dictionary for regulatory activities; TEAEs, treatment emergent adverse events.

[1] n, number of participants reporting event; (%, percentage of total trial population reporting event); m, number of events.

Table S11 Changes in anthropometry and fasting blood samples, from baseline to end of study.

**Variable**<br>**Mean (95% CI)**	**EMP16+fiber**<br>**(N=13)**	**Active control+placebo**<br>**(N=23)**
Relative weight loss	-2.9% (-3.7% to -2.1%)	-1.6% (-2.4% to -0.7%)
Body Mass Index (kg/m^2)	-0.97 (-1.23 to -0.71)	-0.54 (-0.82 to -0.27)
Body fat %	-1.5 (-2.4 to -0.7)	-1.3 (-2.5 to -0.1)
Waist Circumference (cm)	-1.9 (-3.9 to 0.0)	-3.2 (-6.1 to -0.3)
Total cholesterol (mmol/L)	-0.44 (-0.74 to -0.13)	-0.30 (-0.50 to -0.11)
HDL cholesterol (mmol/L)	-0.16 (-0.23 to -0.09)	-0.15 (-0.21 to -0.09)
LDL cholesterol (mmol/L)	-0.44 (-0.70 to -0.17)	-0.36 (-0.49 to -0.23)
Triglycerides (mmol/L)	0.01 (-0.27 to 0.28)	0.32 (0.05 to 0.60)
HbA1c (mmol/mol)	0.54 (-0.09 to 1.16)	0.48 (-0.13 to 1.09)
Glucose (mmol/L)	-0.19 (-0.38 to 0.00)	-0.17 (-0.30 to -0.03)
GGT (uKat/L)	-0.09 (-0.20 to 0.02)	-0.04 (-0.13 to 0.05)
FLI[1]	-3.6 (-6.8 to -0.4)	-1.6 (-5.6 to 2.5)

[1] FLI, Fatty liver index, was calculated from BMI, waist circumference, TGs and GGT [3]); GGT, gamma glutamyl transferase.

Table S12 Post-trial evaluation question "Would you be willing to keep on taking this fibre supplement in the long term?" (FAS)

Number of participants	Yes	Uncertain	No
EMP16+fiber	6	4	3
Active control+placebo	15	4	2

References

1. Lai, H., et al., *Effects of dietary fibers or probiotics on functional constipation symptoms and roles of gut microbiota: a double-blinded randomized placebo trial.* Gut Microbes, 2023. **15**(1): p. 2197837.
2. Major, G., et al., *Demonstration of differences in colonic volumes, transit, chyme consistency, and response to psyllium between healthy and constipated subjects using magnetic resonance imaging.* Neurogastroenterology & Motility, 2018. **30**(9): p. e13400.
3. Bedogni, G., et al., *The Fatty Liver Index: a simple and accurate predictor of hepatic steatosis in the general population.* BMC Gastroenterol, 2006. **6**: p. 33.
